# Supplementary figures and images for: A cryptic K48 ubiquitin chain binding site on UCH37 is required for its role in proteasomal degradation
Source: eLife. 2022 Apr 22;11:e76100. doi: 10.7554/eLife.76100 (PMC9033301; doi:10.7554/eLife.76100)

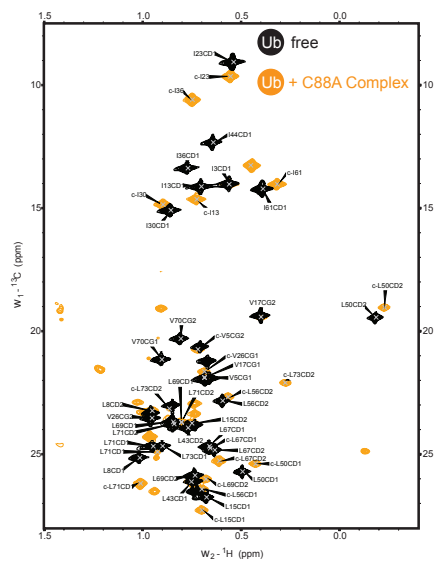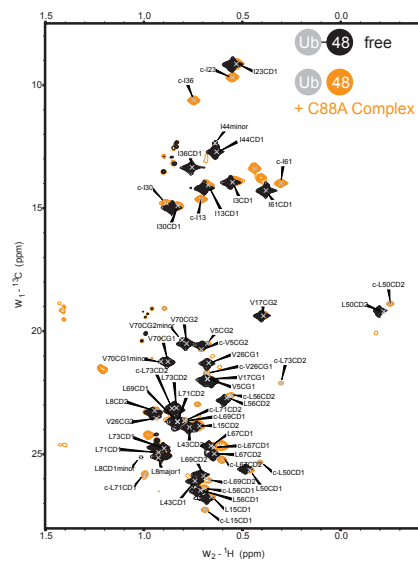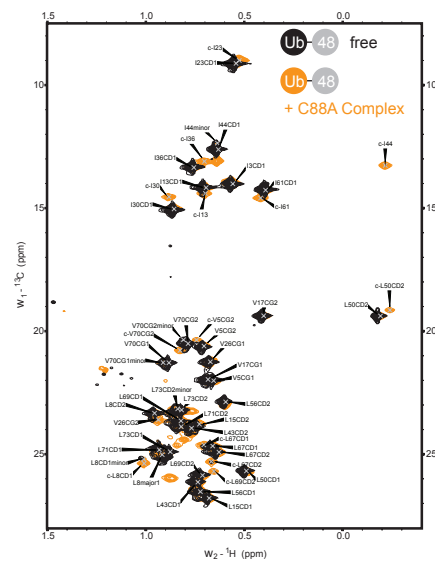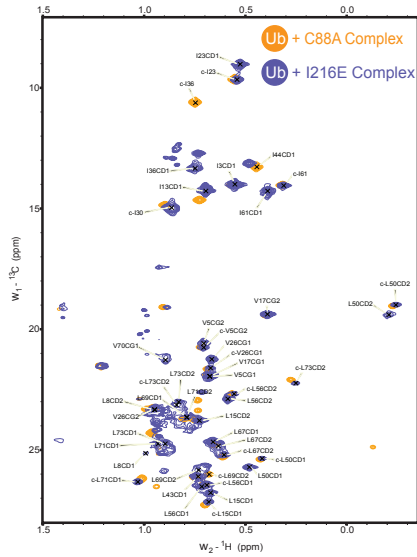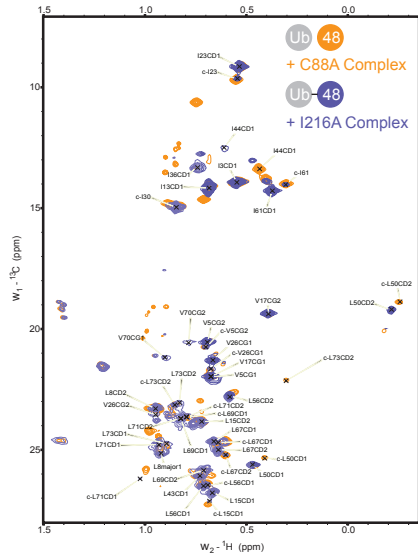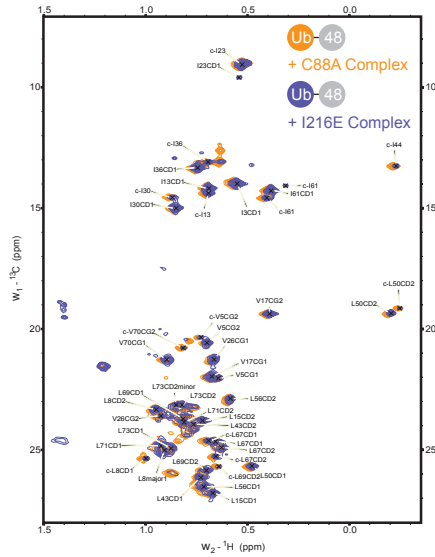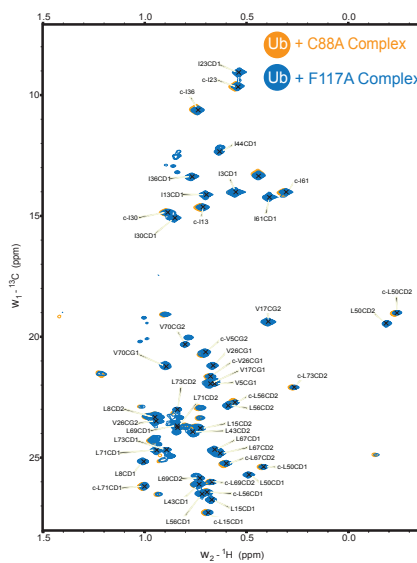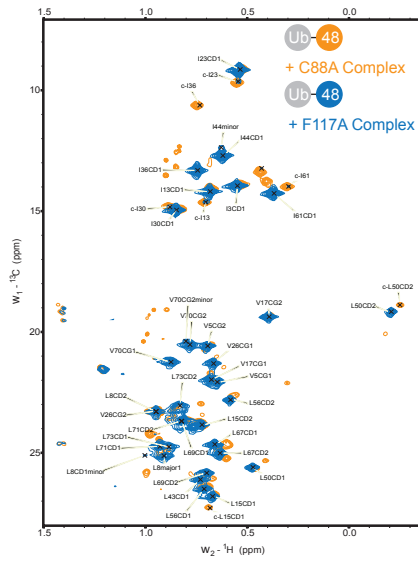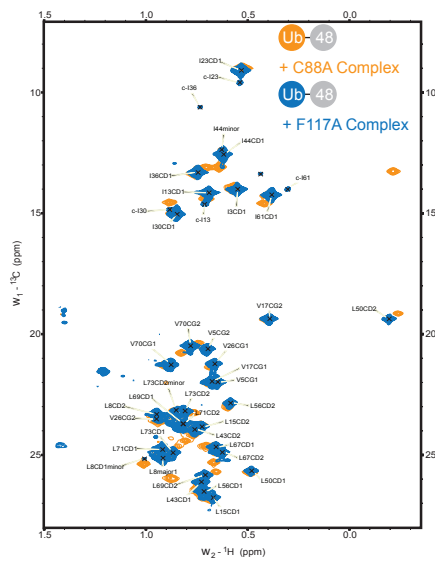

Supplement: Figure 5—source data 1. [file elife-76100-fig5-data1.pdf]
