# Supplementary material for: A cryptic K48 ubiquitin chain binding site on UCH37 is required for its role in proteasomal degradation
Source: eLife. 2022 Apr 22;11:e76100. doi: 10.7554/eLife.76100 (PMC9033301; doi:10.7554/eLife.76100)

Figure 1C

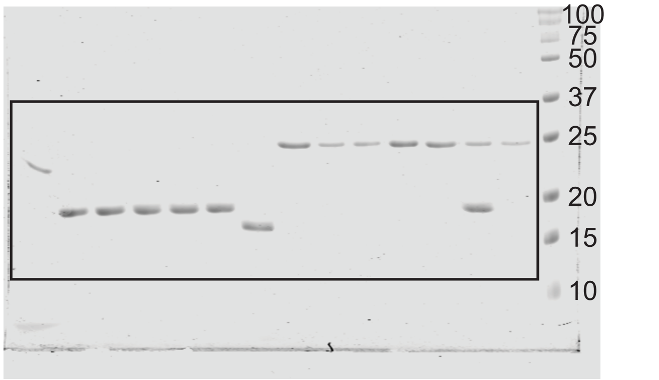

Figure 1D

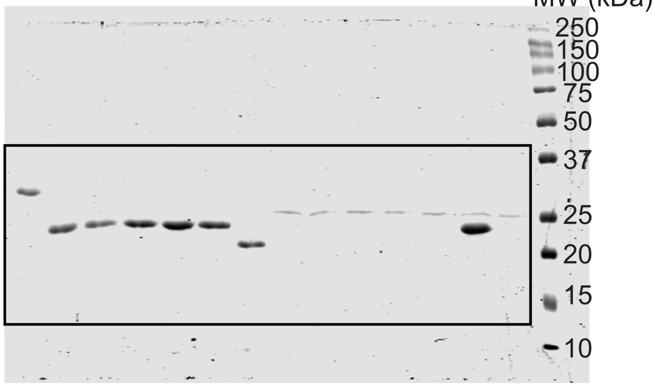

Figure 6B

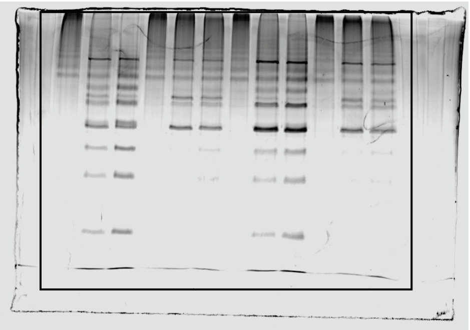

Figure 6B

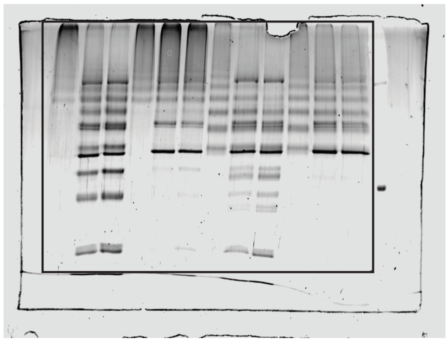

Figure 6B

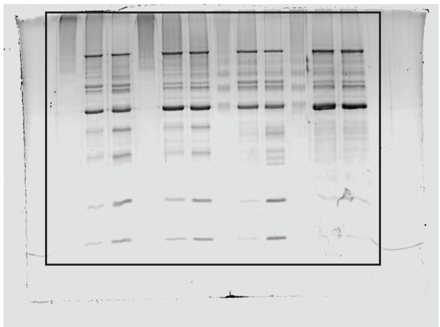

Figure 6C

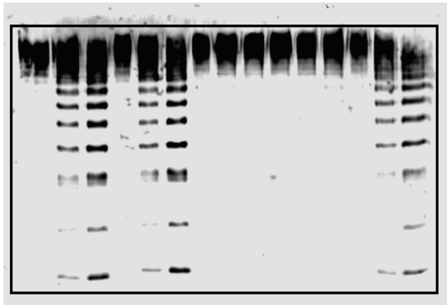

Figure 6D

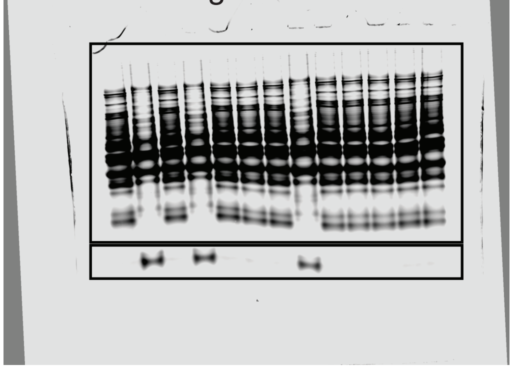

Figure 6D

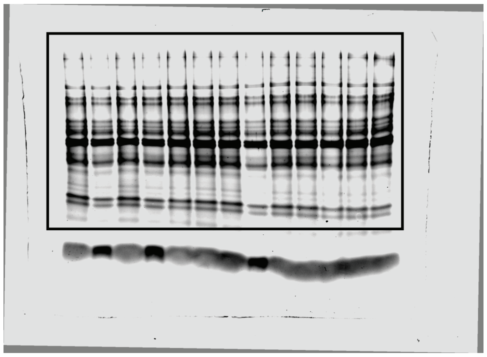

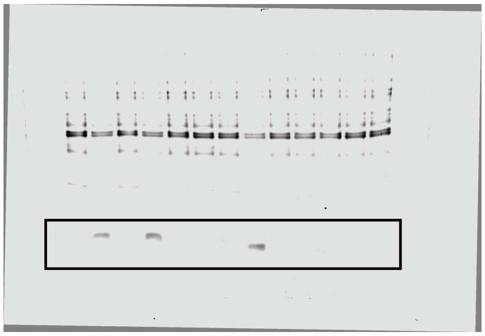

Figure 6I

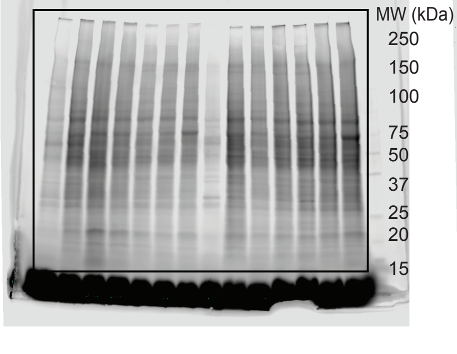

Figure 6I

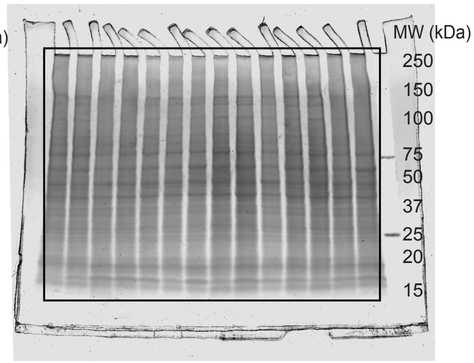

Figure 6E

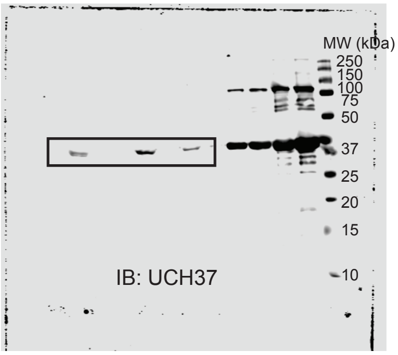

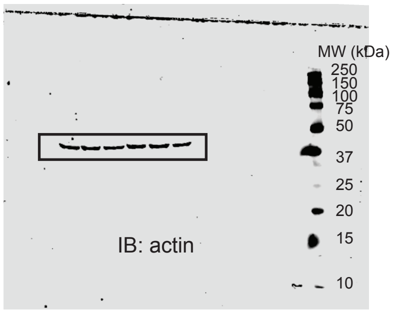

Figure 7E

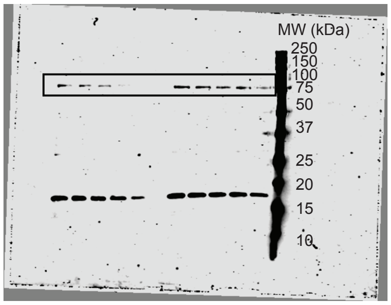

IB: Mfn2

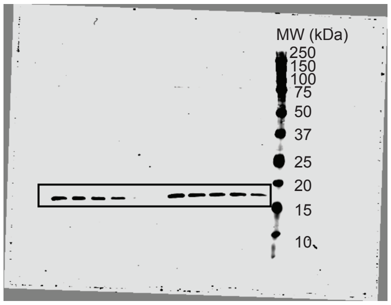

IB: POLR2D

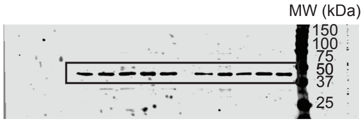

IB:β actin

Figure 7F

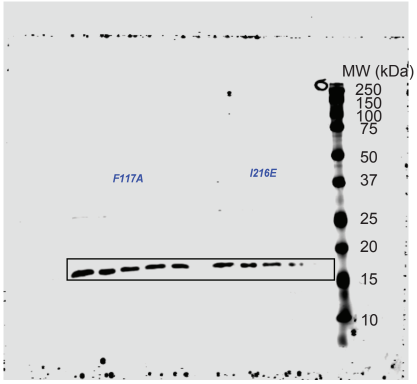

IB: POLR2D

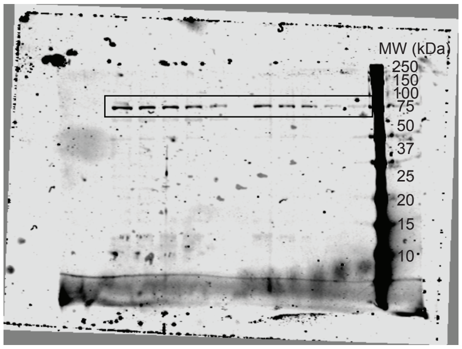

IB: Mfn2

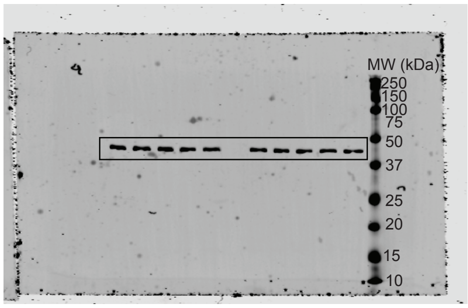

IB:β actin

Figure 7H

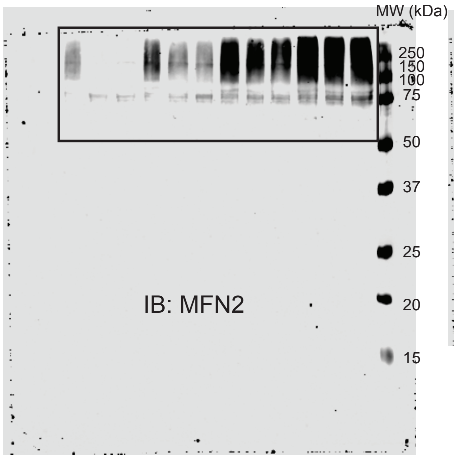

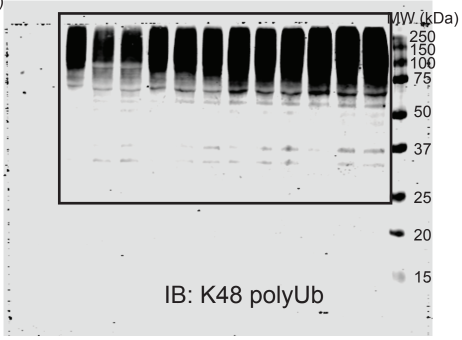

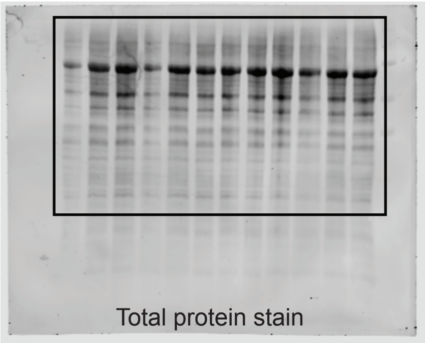

Figure 1 Supplement 1A

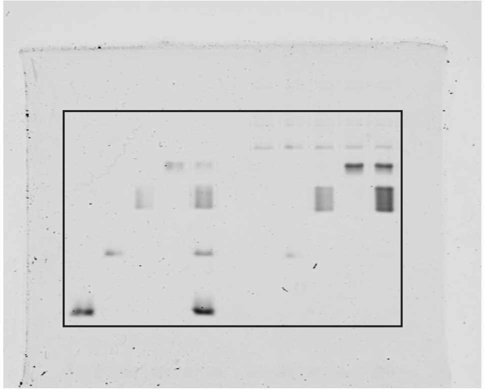

Figure 1 Supplement 1B

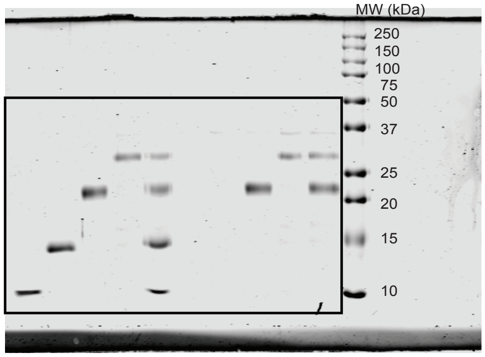

Figure 1 Supplement 1C

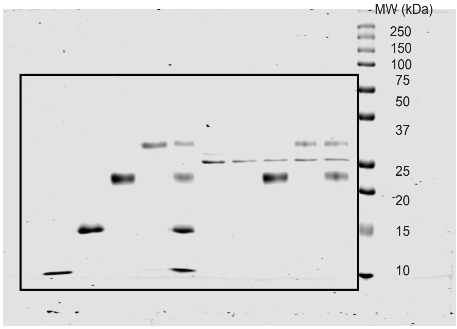

Figure 3 Supplement 1B

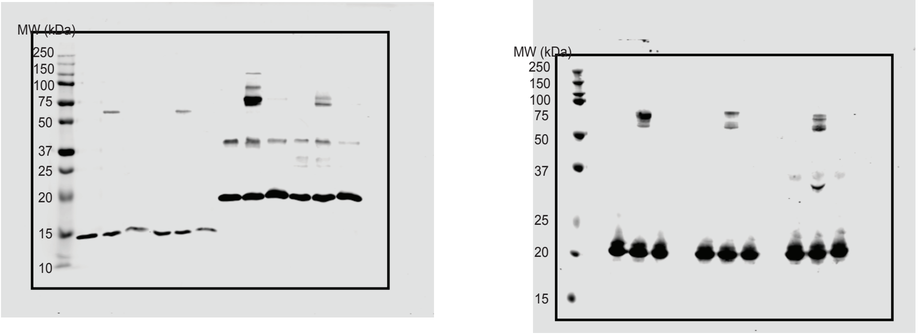

Figure 6 Supplement 1B

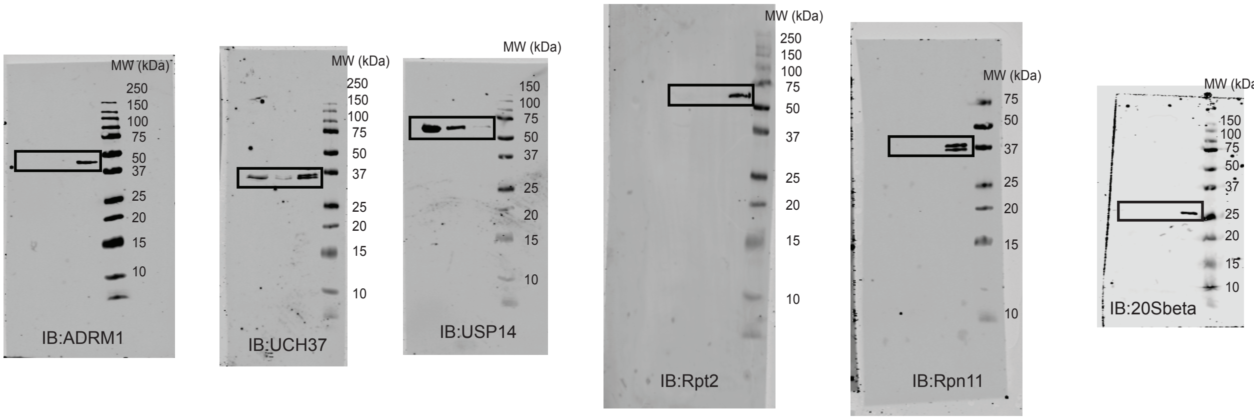

Figure 6 Supplement 1C

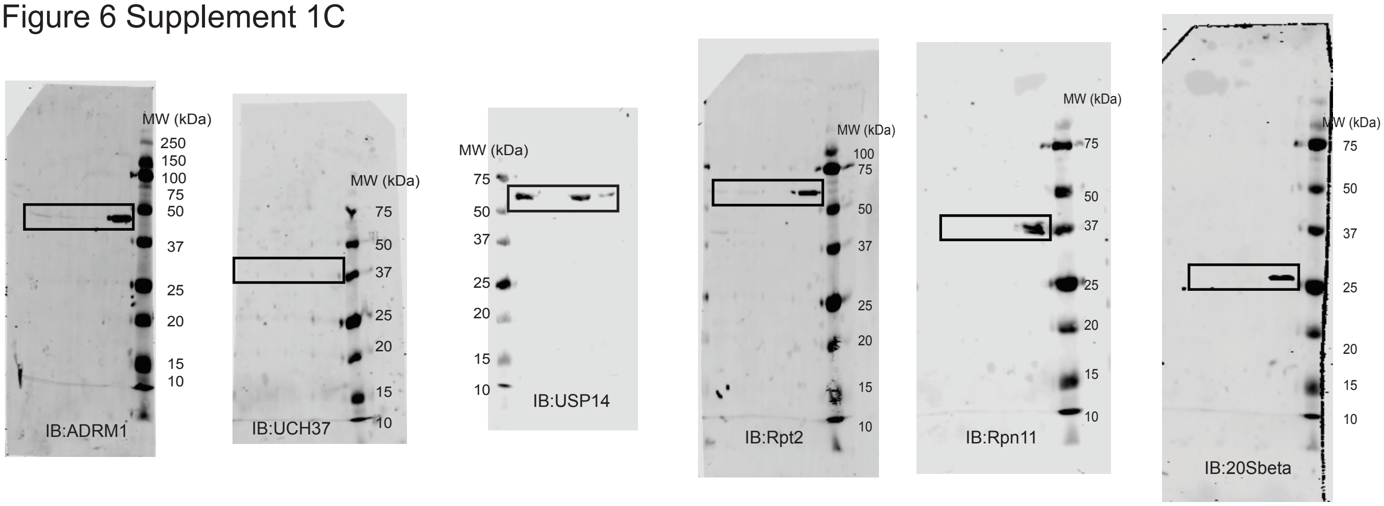

Figure 6 Supplement 1D

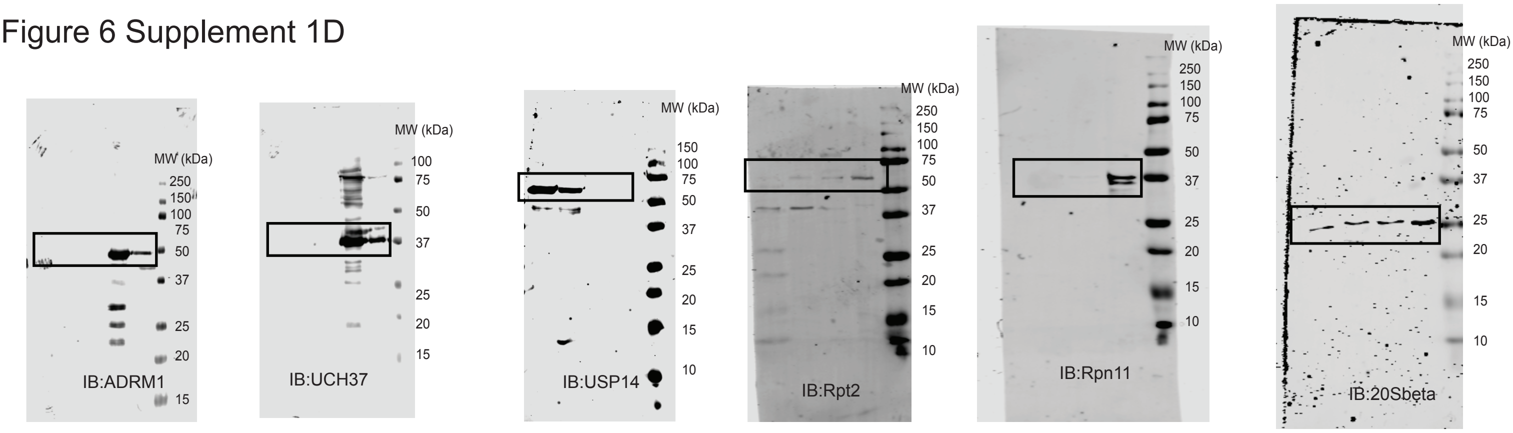

Figure 6 Supplement 1E

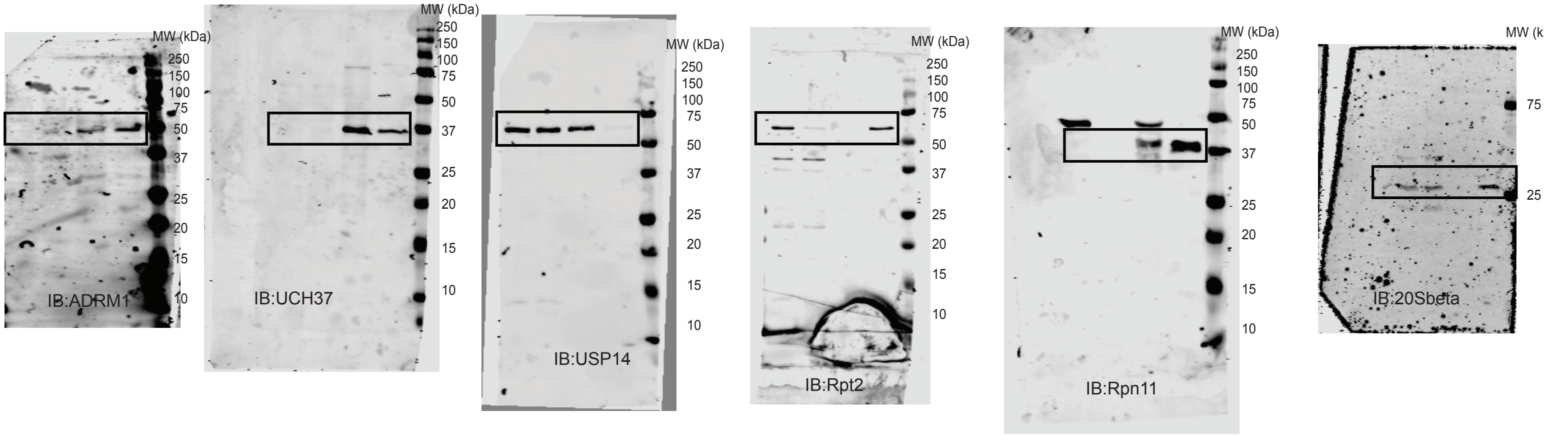

Figure 6 Supplement 1F

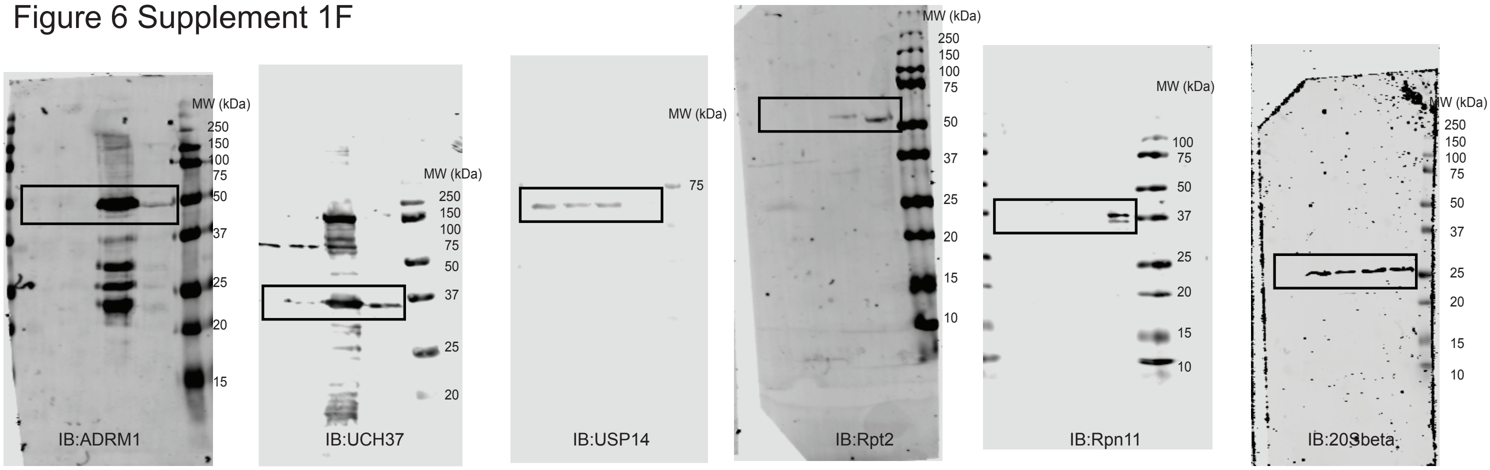

Figure 6 Supplement 1G

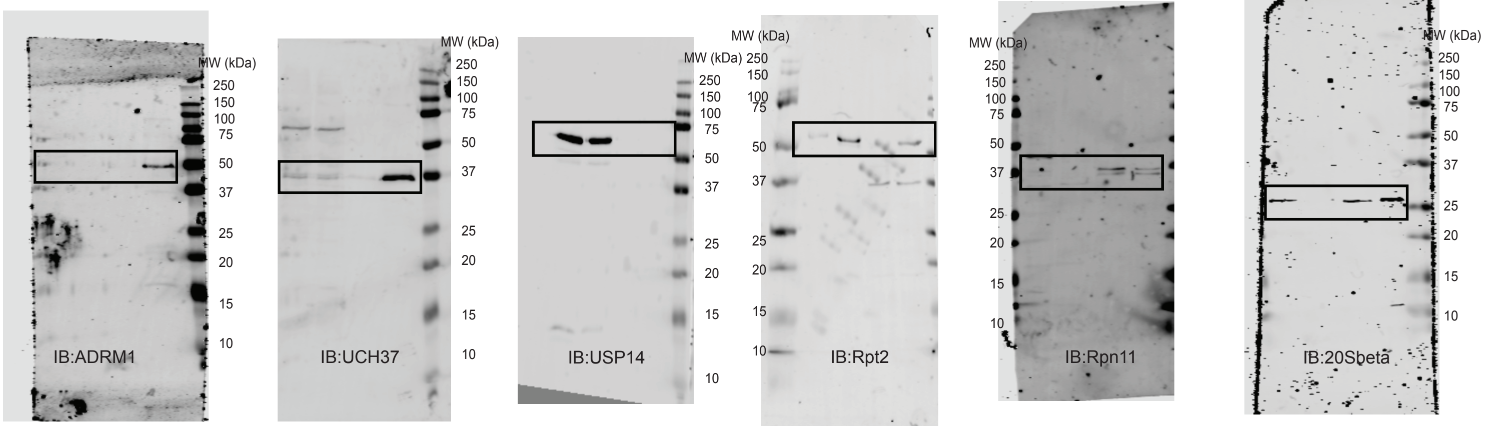

Figure 6 Supplement 1H

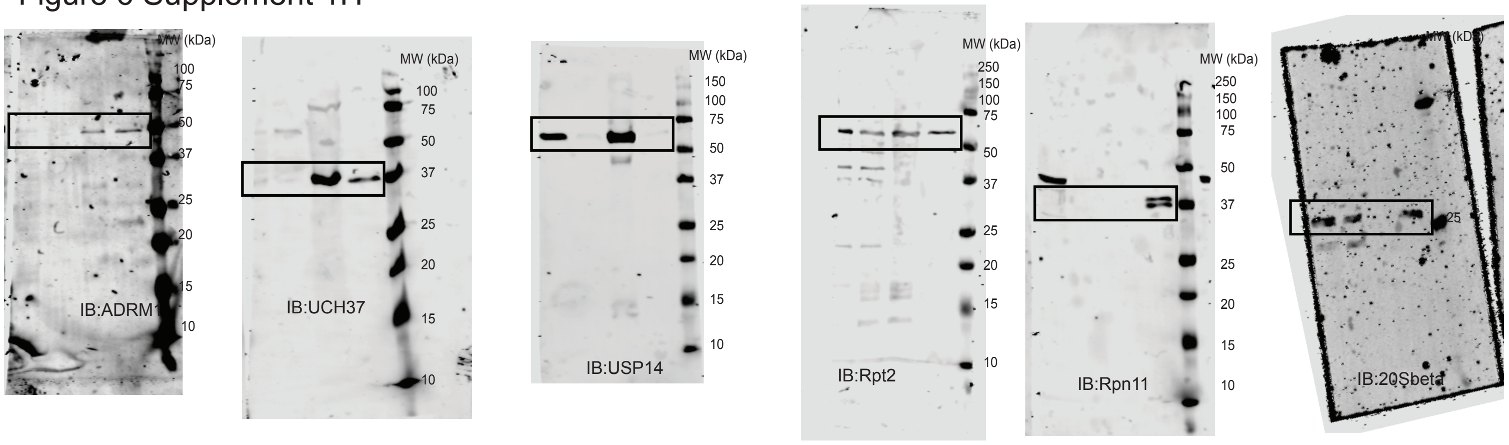

Figure 7 Supplement 1D

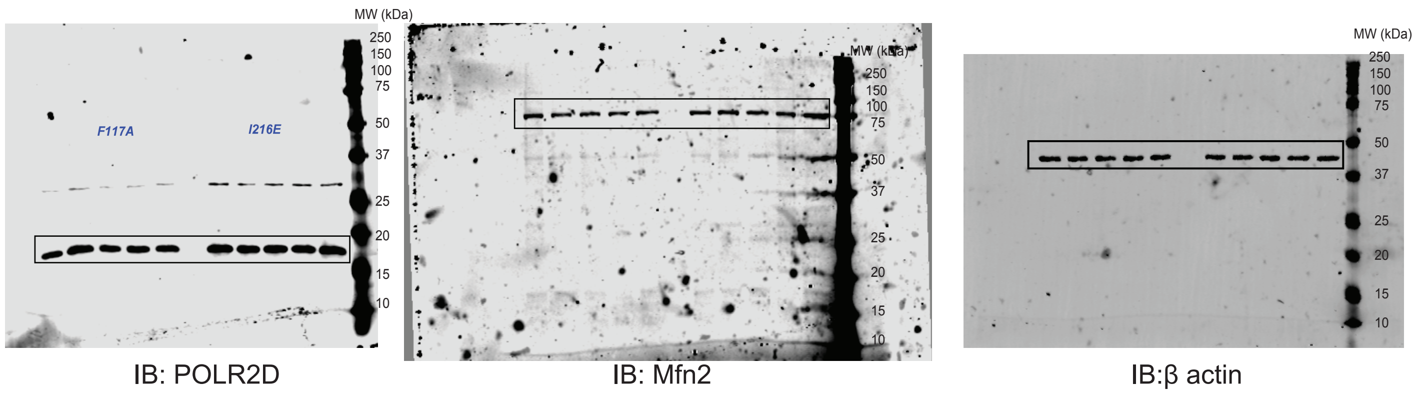

Uncropped kinetic gels for Figure 5

UCH37•RPN13 Kinetics

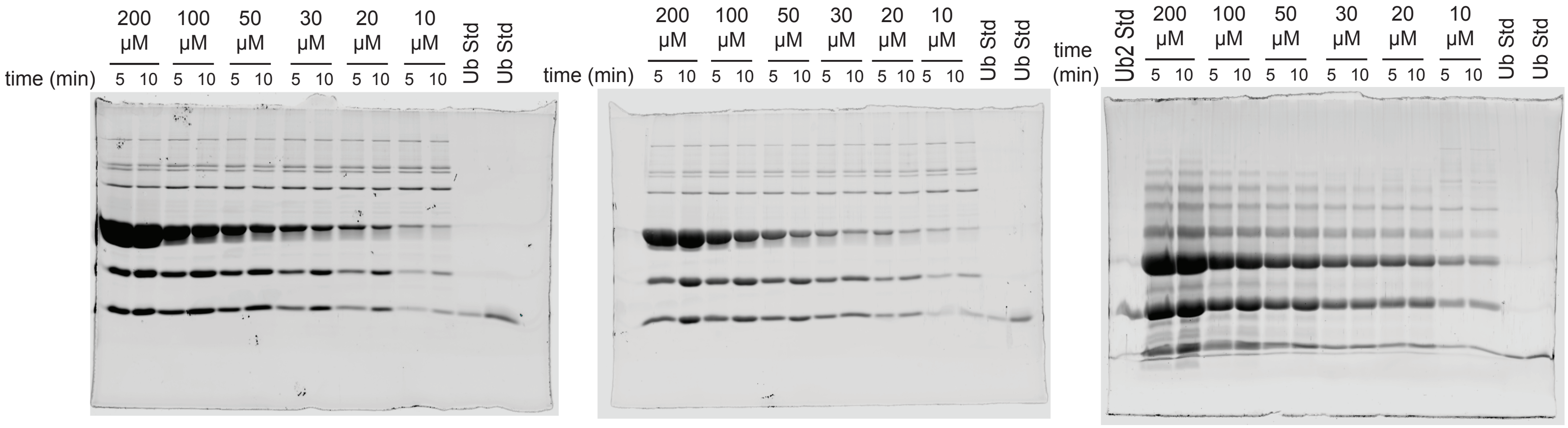

UCH37 I216E•RPN13 Kinetics

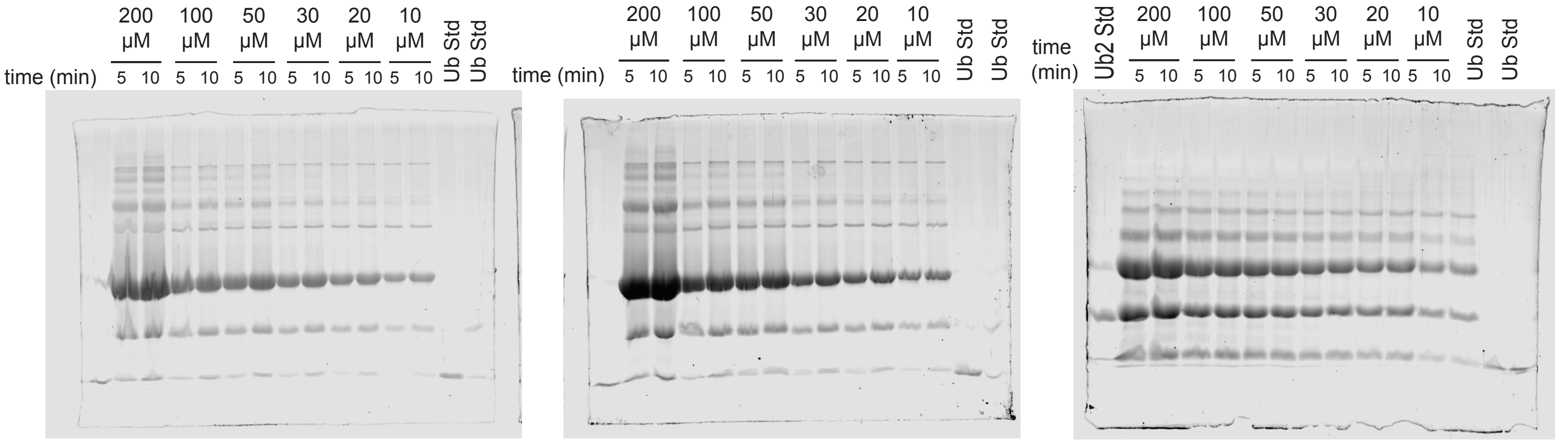

UCH37 L181A•RPN13 Kinetics

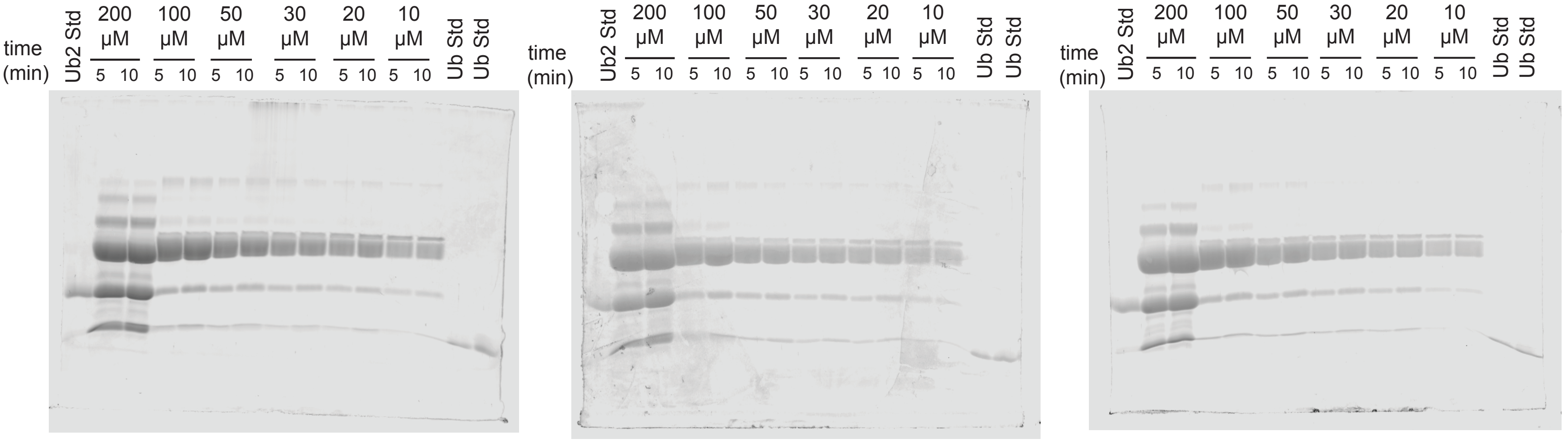

UCH37 F117A•RPN13 Kinetics

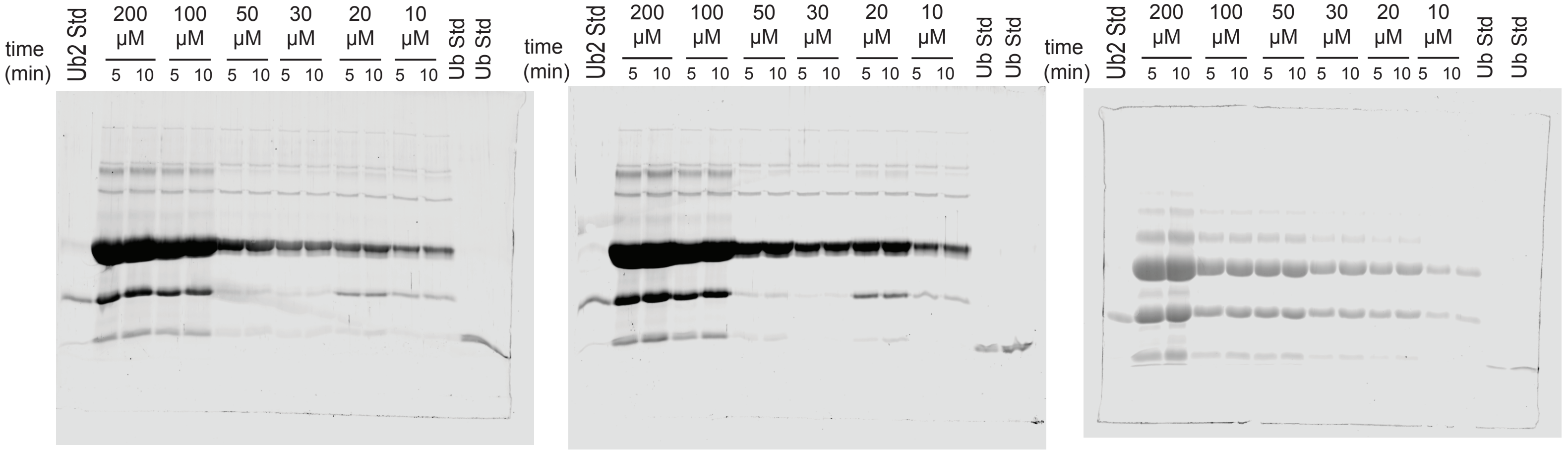

UCH37 F121A•RPN13 Kinetics

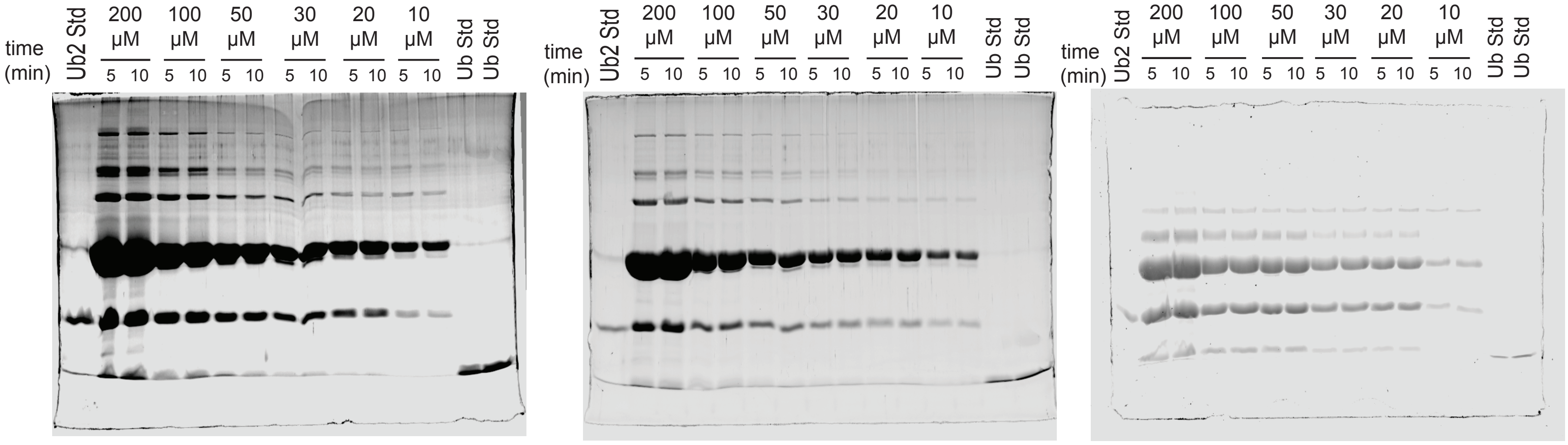

Supplement: Figure 6—source data 2. [file elife-76100-fig6-data2.pdf]
